# Analytical procedure for the determination of very volatile organic compounds (C_3_–C_6_) in indoor air

**DOI:** 10.1007/s00216-018-1004-z

**Published:** 2018-03-28

**Authors:** Alexandra Schieweck, Jan Gunschera, Deniz Varol, Tunga Salthammer

**Affiliations:** Department of Material Analysis and Indoor Chemistry, Fraunhofer Wilhelm-Klauditz-Institut, Bienroder Weg 54E, 38108 Braunschweig, Germany

**Keywords:** VVOC, Indoor air, Analysis, Gas chromatography, Mass spectrometry, Thermal desorption

## Abstract

The substance group of very volatile organic compounds (VVOCs) is moving into the focus of indoor air analysis, facing ongoing regulations at international and European levels targeting on indoor air quality and human health. However, there exists at present no validated analysis for the identification and quantification of VVOCs in indoor air. Therefore, the present study targeted on the development of an analytical method in order to sample the maximum possible quantity of VVOCs in indoor air on solid sorbents with subsequent analysis by thermal desorption and coupled gas chromatography/mass spectrometry (TDS-GC/MS). For this purpose, it was necessary to investigate the performance of available sorbents and to optimize the parameters of GC/MS analysis. Stainless steel tubes filled with Carbograph 5TD were applied successfully for low-volume sampling (2–4 l) with minimal breakthrough (< 1%). With the developed method, VVOCs between C_3_ and C_6_ of different volatility and polarity  can be detected even in trace quantities with low limits of quantitation (LOQ; 1–3 μg m^−3^). Limitations occur for low molecular weight compounds ≤C_3_, especially for polar substances, such as carboxylic acids and for some aldehydes and alcohols. Consequently, established methods for the quantification of these compounds in indoor air cannot be fully substituted yet. At least three different analytical techniques are needed to cover the large spectrum of relevant VVOCs in indoor air. In addition, unexpected reaction products might occur and need to be taken into account to avoid misinterpretation of chromatographic signals.

**Electronic supplementary material:**

The online version of this article (10.1007/s00216-018-1004-z) contains supplementary material, which is available to authorized users.

**Graphical abstract:**
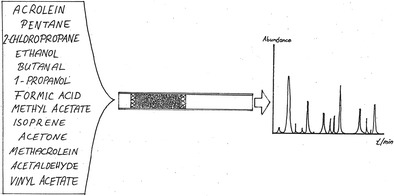
Solid sorbent sampling of VVOCs (C_3_-C_6_) in indoor air with subsequent TDS-GC/MS analysis

## Introduction

Determination of indoor air quality has become of increasing importance against the background of potential adverse effects on human health and well-being due to airborne pollutants [1]. Specific measurement of chemical substances plays a role in many fields of indoor-related research such as sick building syndrome [2], microbial contamination [3], bioeffluents [4], odor evaluation [5], and indoor chemistry [6]. The significance of material emission testing has just recently been outlined by the European Union (EU) Construction Products Regulation (CPR) which defines six basic requirements for construction works (BRCW) [7]. The third basic requirement (BRCW 3) is dedicated to the aspects of hygiene, health, and environment and, therein, points out the protection of the health of building occupants and users as one main target of construction work. Among other things, the “giving-off of toxic gases” and “the emissions of dangerous substances, volatile organic compounds (VOC), greenhouse gases or dangerous particles into indoor or outdoor air” are included. This applies not only to buildings, but also as basic requirement to single materials, products, and furnishing contained in them. Thus, the limitation and prevention of airborne pollutants in indoor environments are explicitly identified and are consequently main conditions regarding the possible release of volatile substances from materials.

The measurement of pollutants indoors has been standardized at the international level in the last decades. The main important standard can be found within the ISO 16000 series targeting on the analysis of organic chemicals in emission test chambers and indoor air [8–10]. On the European level, the performance of chamber emission testing of products used indoors as well as the analysis of organic emissions is harmonized on the basis of EN 16516 [11]. The standard only defines the procedure for testing and chemical analysis, but no harmonized strategy exists so far regarding the evaluation of measured material emissions. In the meantime, national and product-related procedures have been developed in different European countries [12]. Within the European Collaborative Action “Urban Air, Indoor Environment and Human Exposure”, criteria for a harmonized testing procedure and a scheme for a uniform and reproducible health-related evaluation of emissions from building products for indoor use have been derived [13, 14]. The criteria cover VOCs and carbonyl compounds, including formaldehyde [13]. The evaluation is based on the derivation of the so-called LCI (Lowest concentration of interest) levels above which, according to best professional judgment, the pollutant may have some effect on people in the indoor environment [14]. The European work on harmonized EU-LCI values considered in a first stage just VOCs, but with the important note that very volatile organic compounds (VVOCs) should be addressed in the future. In 2013, EU-LCI values for few VVOCs have been published, namely for formaldehyde, acetaldehyde, butanal, and pentanal. With status as of December 2016, the derivation of some other VVOCs is pending (propanal and 2-propanone).

Speaking of VVOCs is linked to the problem that in contrast to VOCs there exists so far neither a uniform definition of the term “VVOC” nor a reliable and robust analytical method for the identification and quantification of many very volatile substances. Salthammer [15] has recently outlined the difficulties and inconsistencies when comparing the different approaches for classifying VVOCs. The European standard EN 16516 [11] defines VVOCs as those substances, which elute before *n*-hexane on a slightly polar gas chromatographic column (5%/95% phenyl-/methylpolysiloxane). However, the standard also offers a normative annex listing noncarcinogenic and carcinogenic VOCs in addition to the analytical window of VOCs (*n*-hexane to *n*-hexadecane). This includes also substances, which can be defined as VVOCs due to their number of carbon atoms (<C_6_), irrespective of whether they are eluting from the gas chromatographic column before or after *n*-hexane [11, 16].

### Analytical approaches

Diverse measurement techniques for the analysis of atmospheric VOC species in outdoor air, including very volatiles such as acetaldehyde, isoprene, and 1,3-butadiene, already exist [17]. The difficulty of retaining very volatiles on solid sorbent tubes when sampling at ambient temperatures can be overcome by collecting whole air samples to pre-evacuated and passivated stainless steel canisters [16, 18]. The method is described in the US EPA Compendium Methods TO-14 and TO-15 [19, 20] as well as in ASTM D5466-15 [21]. Even though the canister sampling technique offers short sampling times, long storage periods of up to 30 days, and low detection limits (1 μg m^−3^), there are several drawbacks. These relate to the repeatability of taken samples which restricts the application of TO-15 to polar substances and organic compounds less volatile than *n*-octane [18]. Condensation, matrix, and sink effects as well as the undesired loss of target substances through the canister walls or during the transfer of the air sample to the analytical device are not totally excluded [16]. In addition, the cleaning and preparation of canisters is extensive and their handling is difficult, and especially time-weighted-average (TWA) samples need a relatively complex apparatus [16]. Moreover, measurement campaigns on-site would require the transport of some dozen canisters.

This might be some of the reasons why most studies have focused on the development of solid sorbent-based methods allowing the application of known analytical steps without the need of additional equipment. The use of porous solid materials for sampling indoor air has become a kind of convention, especially for trapping airborne organic vapors, as it has several main advantages and overcomes serious disadvantages of liquid absorbents [18, 22]. In addition, solid sorbent tubes are easy to store, carry, and transport and are reusable for a specific service life. ASTM D6196-15 [23] offers an extensive guide for air sampling with solid sorbents.

Tenax TA® is a widespread polymer sorbent recommended for retaining VOCs according to ISO 16000-6. It allows the detection and quantification of nearly the most relevant nonpolar and slightly polar substances in the analytical window between *n*-hexane (C_6_) and *n*-hexadecane (C_16_) in one single step. The standard refers to a nonpolar GC column and highlights that the specified method is in principle also suitable for the determination of some VVOCs if appropriate sorbents and adequate gas chromatographic conditions are chosen [8]. However, more detailed specifications are left open. Irrespective of the fact that compounds which might be classified as VVOCs are already collected to a specific extent when sampling indoor air on Tenax TA®, this polymer sorbent is too weak for polar substances. Hence, the quantification of target analytes which are more volatile than *n*-hexane is afflicted with errors [18]. Most studies have therefore investigated graphitized carbon blacks (GCB) and carbon molecular sieves (CMS) as these were introduced as highly sorptive alternatives for retaining reactive or low-boiling hydrocarbons in indoor air while being largely hydrophobic [24–26].

Carbotrap X (20/40 mesh) was found to allow the quantitative determination of low-boiling, reactive hydrocarbons, such as 1,3-butadiene or isoprene with no significant losses of the analytes [25]. Carbograph 5 was also proven to be able to sample low-boiling carbonyl compounds [26, 27]. Dettmer et al. [27] compared both GCBs regarding their adsorption potential of low molecular weight oxygenated substances in gaseous samples. Recovery rates of the analytes were higher for Carbograph 5, despite of the higher specific surface area of Carbotrap X. The recovery might be influenced by relative humidity and the presence of ozone or nitrogen oxides as discussed for the significant losses of reactive light hydrocarbons on CMS [25]. Adsorption of CMS is based on nonspecific interactions with several reaction processes taking place on the adsorbent surface, e.g., the decomposition of α-pinene and sabinene and a dimerization of 1,3-butadiene leading to 4-vinylcyclohexene on Carboxen 569 [25, 28, 29]. Ribes et al. [30] combined GCB and CMS adsorbers in a multisorbent tube in order to analyze a broad range of VOCs in air, targeting especially on isocyanate species. The developed method, based on TDS-GC/MS, allowed also the detection of some small molecular weight compounds, but without carrying out a validation for VVOC analysis or any differentiation of VVOCs from VOCs. Gallego et al. [31] found significant differences between the concentrations obtained from this multibed tube and common Tenax TA® tubes regarding VVOCs with boiling points between 56 and 100 °C and vapor pressures (20 °C) ranging from 4 to 47 kPa. The same was observed for alcohols and chlorinated compounds resulting in higher concentrations obtained by using the multibed tube compared to a Tenax TA® tube. The authors assume that Tenax TA® is not suitable for adsorbing VVOCs due to a displacement of the adsorbed volatile and polar compounds for nonpolar high molecular weight substances, as previously reported [28, 32, 33]. However, this study included just a small range of ten substances defined as VVOCs by boiling point and vapor pressure. In addition, standard deviations from measuring data obtained by indoor air sampling were quite high for most VVOCs when using Tenax TA® as sorbent. Breakthrough volumes for multisorbent bed tubes were low with the exception of ethanol, 2-propanone, dichloromethane, and 2-propanol at high sampling volumes over 40 l. According to Woolfenden [18, 24], tubes containing Tenax TA® backed up by a GCB followed by a CMS should be able to retain C_3_-hydrocarbons up to long-chained alkanes. However, when handling CMS, water management becomes an important issue [34, 35]. Brown and Crump [36] determined the breakthrough volume of six VVOCs (mainly C_4_–C_6_ alkanes) on such a multisorbent tube resulting in sample volumes of at least 10 l.

Facing the current approaches and challenges, the aim of the present study was to develop an analytical procedure to measure concentrations of VVOCs in indoor air. For this purpose, it was necessary to determine the performance of different solid sorbents for their sorption/desorption capacities of VVOCs, to develop a suitable GC/MS method, and to consider already established techniques.

## Materials and methods

### Sorbent selection and conditioning

The selection of sorbent materials was based on their chemical and physical material properties as well as on previous studies. As the target substances to be investigated within this study are very volatile organics with low boiling points, mainly medium and strong sorbent media of different mesh sizes were selected. The mesh size indirectly determines the particle size and, hence, the specific surface area of the solid sorbent. Among other parameters, the surface area is one important factor describing the sorbent strength of the material. According to Woolfenden [24], the mesh size within the 30–80 range does not play a critical role regarding selection of solid sorbents as the analyte retention volume will remain constant. Patil and Lonkar [37] investigated Tenax TA® of different mesh sizes for sampling volatile organic species in workplace air. No significant effects on adsorption and desorption in dependence of the particle size were found. Materials chosen in the presented study comprised the GCBs Carbotrap (20/40 mesh, Sigma-Aldrich), Carbopack X (40/60 mesh, Sigma-Aldrich), and Carbograph 5TD (20/40 mesh, Markes International Ltd.) as well as the CMS Carbosieve S-III (60/60 mesh, Supelco), Carboxen 569 (20/45 mesh, Supelco), and Carboxen 1000 (80/100 mesh, Supelco). Table 1 gives an overview of their relevant properties. For further information on GCBs and CMS, please refer to the literature [24, 28, 35].

**Table 1:** Chemical and physical properties of selected adsorbent materials. Data provided by the manufacturers and Dettmer and Engewald [28]

Adsorbent type	Adsorbent	Particle size [mesh]	*T*_max_ [°C]	*T*_des_ [°C]	Density [g ml^−1^]*	Pore volume [ml g^−1^]*		Specific surface area [m^2^ g^−1^]*	Sorbent strength	Substance range	Notes
						Micro	Total				
Graphitized carbon black (GCB)	Carbotrap	20/40	> 400	300–350	0.36	–	0.58	100	Medium/weak	C_5/6_–C_14_	Hydrophobic
	Carbopack X	40/60	> 400	300–350	0.41	0.0	0.63	250	Medium	C_5/6_–C_9_	Hydrophobic
	Carbograph 5 TD	20/40	> 400	300–350	–	–	–	560	Medium	C_5_–C_8_	Hydrophobic
Carbonized molecular sieve (CMS)	Carbosieve S-III	60/80	> 400	300–350	0.61	0.38	0.39	820	Very strong	C_2_–C_5_	Some hydrophilicity; methanol is retained
	Carboxen 569	20/45	> 400	300–350	0.58	0.07	0.39	485	Very strong	C_2_–C_5_	Some hydrophilicity; methanol is retained
	Carboxen 1000	80/100	> 400	300–350	0.44^a^	0.42^a^	0.85^a^	> 1200	Very strong for small molecules	C_2_–C_3_	Significant hydrophilicity; methanol is retained

*Manufacturer data

^a^Data given for Carboxen 1000 (60/80 mesh)

In order to prepare single-bed tubes, ~ 300 mg of the selected sorbents were placed in stainless steel desorption tubes (Markes International Ltd., 89 mm length, 6.4 mm O.D.) between glass wool end plugs. Initial conditioning of freshly packed tubes was performed at 300 °C for 3 h in total, whereas heating was done at different stages during preparation of the tubes. Before each use, all tubes were conditioned for 115 min at a maximum temperature of 300 °C under a helium flow. After conditioning, tubes were immediately sealed using Swagelok brass end caps fitted with PTFE ferrules and stored in closed metal boxes. Sampled tubes were desorbed and analyzed immediately after finishing the tests in order to avoid analyte losses due to storage time [25].

### Chemicals

Target substances used in this study were selected based on the elution time before *n*-hexane on a nonpolar or slightly polar GC column. They can therefore not be determined by the procedure given for VOCs (C_6_–C_16_) in ISO 16000-6 [8]. Table 2 summarizes the selected organic compounds and their specific properties. Chemicals were purchased from Sigma-Aldrich with purities of ≥ 99%. Absolute grade methanol used for the preparation of the standard solution was supplied by Sigma-Aldrich.

**Table 2:** Properties of method target analytes eluting before *n*-hexane on a nonpolar or slightly polar GC column: (i) number of carbon atoms (C_*n*_), (ii) molecular weight (MW), and (iii) boiling point (b.p.) [38]

C_*n*_	Compound	CAS no.	Formula	MW [g mol^−1^]	b.p. [°C]
C_1_	Formic acid	64-18-6	HCOOH	46.0	101
C_2_	Ethanol	64-17-5	C_3_H_5_OH	46.1	78.3
	Acetaldehyde	75-07-0	CH_3_CHO	44.1	20.8
	Acetic acid	64-19-7	CH_3_COOH	60.1	118
C_3_	1-Propanol	71-23-8	CH_3_(CH_2_)_2_OH	60.1	97.2
	2-Propanol	67-63-0	CH_3_CH(OH)CH_3_	60.1	82.3
	Propanal	123-38-6	C_3_H_6_O	58.1	48.0
	2-Propanone	67-64-1	CO(CH_3_)_2_	58.1	56.1
	Methyl acetate	79-20-9	CH_3_COOCH_3_	74.1	56.8
	2-Chloropropane	75-29-6	CH_3_CHClCH_3_	78.5	35.0
	Trimethylsilanol	1066-40-6	C_3_H_10_OSi	90.2	99
C_4_	*n*-Butanal	123-72-8	C_4_H_8_O	72.1	74.8
	2-Methylpropanal	78-84-2	CH_3_CH(CH_3_)CHO	72.1	63.5
	2-Methyl-2-propanol	75-65-0	CH_3_C(CH_3_)(OH)CH_3_	74.1	82.9
	Methacroleine	78-85-3	CH_2_C(CH_3_)CHO	70.1	72.9
	Methyl vinyl ketone	78-94-4	CH_3_C(O)CHCH_2_	70.1	81.4
	Vinyl acetate	108-05-4	CH_3_C(O)OCHCH_2_	86.1	71.6
C_5_	*n*-Pentane	109-66-0	C_5_H_12_	72.2	36.1
	Isoprene	78-79-5	CH_2_C(CH_3_)CHCH_2_	68.1	34.0
C_6_	3-Methylpentane	96-14-0	C_2_H_5_CH(CH_3_)C_2_H_5_	86.2	63.3

### Standard solution

The standard solution was prepared as mixture of all selected VVOCs (see Table 2) by weighing 10 mg of each substance into a glass flask, which was filled up with 10 ml methanol to obtain a standard concentration of 1 mg ml^−1^ of each substance.

### Analysis of VVOCs

Analysis of target substances designated as VVOCs was performed by automatic thermal desorption (TD-100, Markes International Ltd.) with subsequent capillary gas chromatography (Agilent 7890A) coupled with a mass spectrometry detector (Agilent 5975C). Conditions for thermal desorption were used as follows: prepurge 3 min at a flow rate of 50 ml min^−1^, primary desorption at 300 °C for 6 min with a flow rate of 20 ml min^−1^, no inlet split, cold trap low 25 °C, pretrap fire purge 3 min at 50 ml min^−1^, trap heating rate 40 °C s^−1^, cold trap high at 300 °C for 6 min, outlet split 10 ml min^−1^, and flow path temperature at 200 °C. The cold trap contained quartz wool/Carbograph 1TD (40/60 mesh) and Carboxen 1000 (80/100 mesh) with a ratio of 1:4.

The GC was fitted with a fused silica capillary column of medium polarity (DB 624, 60 m, 0.32 mm, 1.8 μm, Agilent (J&W); composition 6%/94% cyanopropylphenyl/dimethylpolysiloxane). The column oven temperature was initially 30 °C for 6 min, increased in a first step to 40 °C at a rate of 1 °C min^−1^, in a second step to 70 °C at a rate of 5 °C min^−1^, and maintained after a third increasing rate of 20 °C min^−1^ at 240 °C for 10 min (40.5 min run). The GC was operated in scan mode with a mass range of 20–450 amu, MS source temperature 230 °C, and quadrupole temperature 150 °C.

Data were processed using ChemStation® software mass spectral library. Qualifying was based on PBM library search [39]. Mass spectra and retention data were compared with those of reference compounds. All identified substances were quantified using their own response factors.

### Calibration

The limit of detection *x*_LOD_ and limit of quantitation *x*_LOQ_ for each target analyte were calculated from the linear calibration curve *y* = *a* ∙ *x* + *b*. Calculation was based on the approach given by Einax et al. [40] with reference to DIN 32645 [41]:

1 $$ {x}_{\mathrm{LOD}}={s}_{x0}\bullet {t}_{f;\alpha}\sqrt{\frac{1}{m}+\frac{1}{n}+\frac{{\overline{x}}^2}{Q_x}} $$

*s*_*x*0_ is the standard deviation of the method, *t*_*f*; *α*_ is the *t* -value for *f* degrees of freedom and an error probability *α*, and *k* is a conventional factor to weigh the uncertainty of the result and is usually set to *k* = 3 for an uncertainty of 33.3% [40, 41]. A one-sided *t* -test was applied for *f* = *n* − 2 and *α* = 0.01 as significance level (99%). *n* is the number of calibration points *x*_*i*_, and *m* is the number of samples measured of each concentration *x*_*n*_ − *x*_*n* + 1_. ${\overline{x}}^2$ is the square of the arithmetic mean of the content of all calibration samples and *Q*_*x*_ is the sum of quadratic deviations of *x*. The limit of quantitation *x*_LOQ_ was obtained from Eq. (2).

2 $$ {x}_{\mathrm{LOQ}}=k\cdotp {s}_{x0}\kern0.5em {t}_{f,\alpha}\sqrt{\frac{1}{m}+\frac{1}{n}+\frac{{\left({x}_{\mathrm{LOQ}}-\overline{x}\right)}^2}{Q_{\mathrm{x}}}} $$

Equation (2) is recursive starting with *x*_LOQ_ = *k* ∙ *x*_LOD_ . The standard deviation of the method *s*_*x*0_ is calculated from Eq. (3).

3 $$ {s}_{x0}=\frac{s_{y,x}}{b} $$

*s*_*y*, *x*_ is the residual standard deviation of the calibration measurement values, and *b* is the slope of the linear calibration curve.

Limits of detection *x*_LOD_ and limits of quantitation *x*_LOQ_ obtained for each VVOC target analyte are discussed at the end of the method development part. Calculation details are given in the “Limits of detection and limits of quantitation” section.

## Experimental results and discussion

### Gas chromatographic selectivity for target analytes

**Fig. 1:**
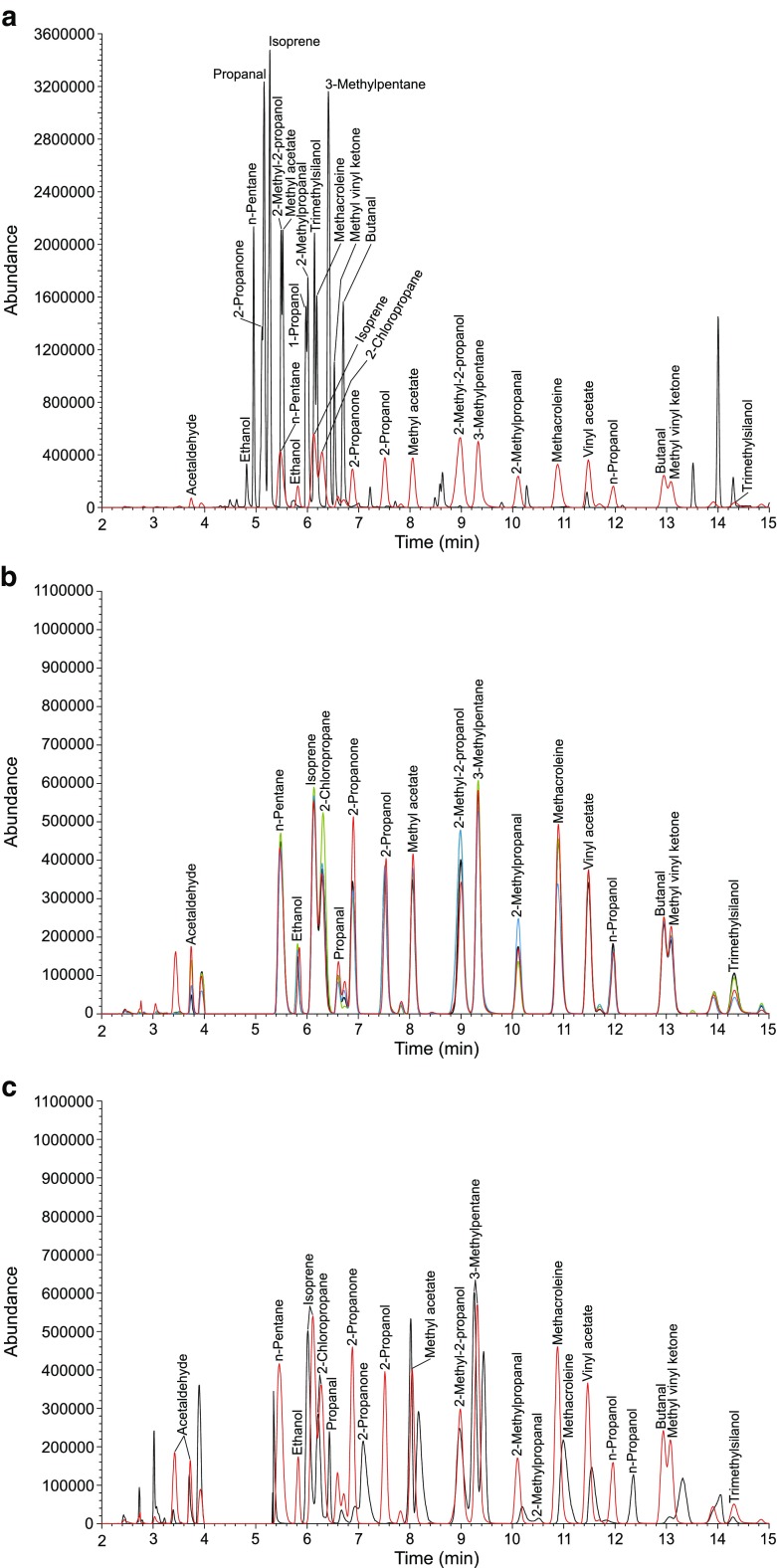
**a** Gas chromatographic (GC) separation of a VVOC standard solution injected on and thermally desorbed of Tenax TA® on a nonpolar GC column (black plot; DB 5, 60 m × 0.25 mm × 0.25 μm) and on a medium polar GC column (red plot; DB 624, 60 m × 0.32 mm × 1.8 μm). Total ion chromatogram (TIC). **b** Gas chromatographic (GC) separation of a VVOC standard solution injected on and thermally desorbed of Carbotrap (black plot), Carbopack X (green plot), Carbograph 5TD (red plot), and Tenax TA® (gray plot). Medium polar GC column (DB 624, 60 m × 0.32 mm × 1.8 μm). Total ion chromatogram (TIC). **c** Gas chromatographic (GC) separation of a VVOC standard solution injected on and thermally desorbed of the CMS Carboxen 569 (black plot) and the GCB Carbograph 5TD (red plot). Medium polar GC column (DB 624, 60 m × 0.32 mm × 1.8 μm). Total ion chromatogram (TIC). **b** and **c** Detector was switched off in the retention window of methanol (4.00–5.30 min), which was used as solvent for the standard solution

An aliquot of 1 μl of the standard solution was injected on three tubes of each adsorbent media including Tenax TA®. Directly after injection, the tubes were analyzed by TDS-GC/MS as described in the “Analysis of VVOCs” section. As shown in Fig. 1a, a VVOC substance mixture, injected on Tenax TA®, cannot be sufficiently separated on a nonpolar gas chromatographic column according to ISO 16000-6 [8]. The target analytes elute in a narrow window, most of them are co-eluting or overlapping. Modifications of the complete analytical setup are necessary in order to obtain bell-shaped peaks (Gaussian curves) and a satisfying separation. Needed changes cover both the thermal desorption and gas chromatographic system and, in particular, the cooling trap, the GC column, and the temperature programs.

**Fig. 2:**
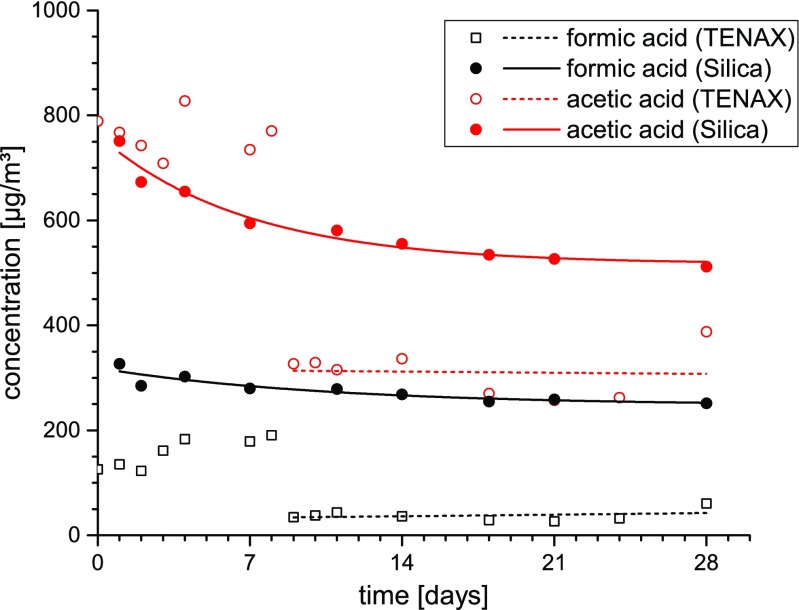
Comparison of formic acid and acetic acid concentrations obtained by active sampling on Tenax TA® (TDS-GC/MS) and on silica gel (IC) during chamber emission testing of a building product over 28 days

After adjustment of these parameters, the gas chromatographic separation of target analytes can be significantly improved, even though the analytes were injected again on a Tenax TA® tube (see Fig. 1a). The use of a stronger adsorbent material, which is more convenient for low-boiling substances, leads in a second step to an increased analyte-adsorbent interaction and, thus, to a further improvement. Especially concerning the tested GCB adsorbent media, the gas chromatographic separation achieved is satisfactory and the analytical window is broadened. As can be seen in Fig. 1b, response and separation performance of VVOC analytes on a medium polar GC column are the best after thermal desorption from Carbograph 5TD in comparison to other GCB adsorbents. Gas chromatographic separation in dependence of the solid sorbent decreased in the following order: Carbograph 5TD > Carbopack X > Carbotrap > Tenax TA®. When using CMS as sorbent bed, neither exploitable peak forms nor a sufficient separation of single peaks could be obtained. Retention times of target analytes were strongly shifted compared to those obtained when applying GCBs (see Fig. 1c). Regardless of the chosen sorbent, both formic acid and acetic acid eluted with a strong tailing. Moreover, the signal of formic acid can hardly be distinguished from the background. This finding shows that neither the investigated solid sorbents nor the analytical procedure is appropriate for detecting formic acid and acetic acid. It is therefore recommended to analyze both substances by ion chromatography after trapping on pretreated silica gel-filled cartridges as recently standardized in VDI 4301-7 [42]. After elution with sodium carbonate solution, the compounds are separated on an anion separation column coupled with a conductivity detector. The method allows a much more precise determination of C_1_–C_2_ carboxylic acids in indoor air as by the use of Tenax TA® and subsequent analysis by TDS-GC/MS as it is currently common practice (see Fig. 2).

### Adsorption performance for target analytes

**Fig. 3:**
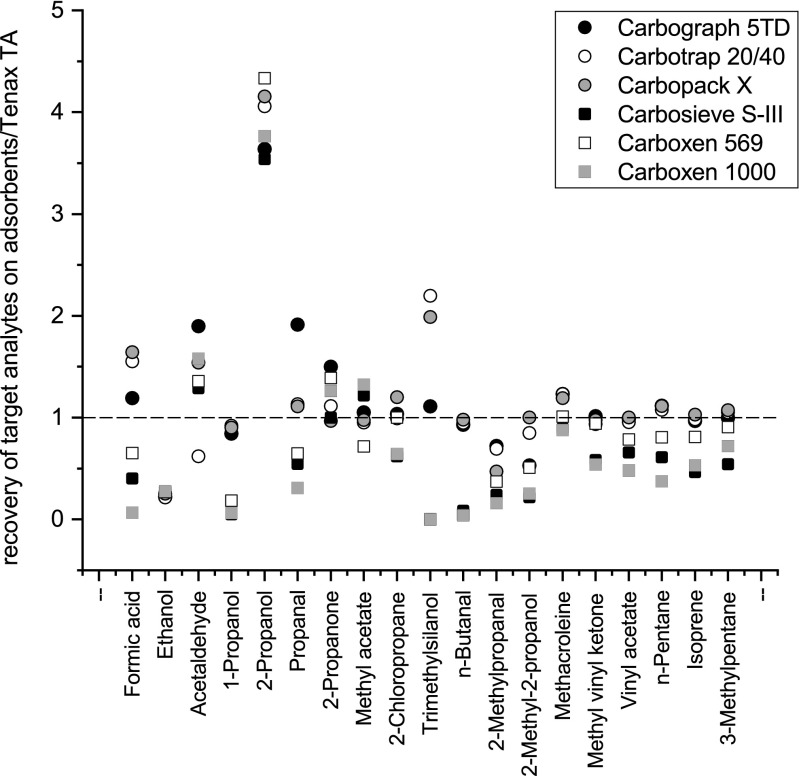
Recovery of selected target analytes on tested solid adsorbents in relation to recovery rates on Tenax TA®. Results are standardized to Tenax TA® = 1

The adsorption performance of different adsorbent materials for selected target analytes can be described by recovery rates. Again, an aliquot of 1 μl of the standard solution was injected on three tubes of each solid sorbent including Tenax TA®. Target analytes have to be equally well adsorbed by the sorbent bed and desorbed again in the subsequent thermal desorption step. The recovery rates were calculated by standardizing the arithmetic mean of the peak areas of each adsorbent media to the arithmetic mean of Tenax TA® obtained by triple measurements. Tenax TA® was chosen as reference even though the authors are aware that it is not suitable for VVOCs. The arithmetic mean of each target analyte obtained by Tenax TA® was set to 1. Sorbents more suitable for retaining VVOCs are characterized by calculated data higher than 1. Values lower than 1 can be traced back either to weaker adsorption performances or to a very strong adsorption which impedes the desorption process (see Fig. 3).

Recovery rates for CMS were below 1. As an exception, 2-propanone, methyl acetate, and acetaldehyde were both well adsorbed and desorbed. Carboxen 569 was also able to sample methacroleine, chloropropane, methyl vinyl ketone, and 3-methylpentane with recovery values between 0.9 and 1. Most of those substances which have been adsorbed well on CMS sorbents were low molecular weight substances with two and three carbon atoms, even though not all small molecules were adsorbed and desorbed equally well. Results for formic acid were again poor. Recovery of 3-methylpentane on Carboxen 569 was satisfying, although this is a C_6_ compound with a relatively high molecular weight (86.2 g mol^−1^).

The tested GCB adsorbent materials showed good recoveries for nearly all target analytes. The best adsorption performances were achieved by using Carbopack X and Carbograph 5TD, respectively, which were superior to Carbotrap. The lowest recovery rates were found for ethanol and 2-methylpropanal even though these two substances differ regarding the number of carbon atoms and physical characteristics. Furthermore, recovery rates for *n*-butanal were not superior to Tenax TA®. For all sorbents tested, recoveries higher than 3.5 were found for 2-propanol, which cannot be reasonably explained.

The results indicate that GCB-filled tubes are able to adsorb a broad range of low molecular substances (C_3_–C_6_) with some limitations concerning alcohols and aldehydes. For compounds ≤C_3_, CMS appear to be the better sampling media. Low molecular weight carbonyl compounds in air are recommended to be analyzed according to ISO 16000-3 [43] after derivatization with 2,4-dinitrophenylhydrazine (DNPH) and subsequent separation and detection by HPLC/UV.

### Breakthrough and safe sampling volumes

For determining the breakthrough and the sampling reproducibility, two sampling tubes of each adsorbent were connected in series. The first tube was spiked with an aliquot of 1 μl of the VVOC standard solution. The exit of the back-up tube was connected with a calibrated sampling pump. The tube pairs were subjected to three different flow rates and two sampling volumes: (a) 50 ml min^−1^, 2 l; (b) 125 ml min^−1^, 2 l; and (c) 125 ml min^−1^, 4 l. Table 3 gives the arithmetic mean and the average standard deviations of the breakthrough, obtained by double measurements. Breakthrough is given as % VVOC (target analyte) in the back-up tube. Sampling reproducibility was evaluated by calculating the relative standard deviation (%) of the duplicates. Table 3 does not include data for Carboxen 1000, which was representatively tested as CMS adsorbent. The thermal desorption and gas chromatographic separation of target analytes was poor so that the obtained chromatograms showed no sharp peak form and could not be therefore evaluated, especially regarding the back-up tube.

**Table 3:** Breakthrough volumes for analyte/adsorbent combinations (% VVOC found in the back-up tube in relation to the sum of the first and second tube)

C_*n*_	Compound	2 l				2 l				4 l			
		[50 ml min^−1^]				[125 ml min^−1^]				[125 ml min^−1^]			
		Tenax TA^®^	Carbograph 5TD	Carbopack X	Carbotrap	Tenax TA^®^	Carbograph 5TD	Carbopack X	Carbotrap	Tenax TA^®^	Carbograph 5TD	Carbopack X	Carbotrap
C_1_	Formic acid	9.72 ± 8.59	0.00 ± 0.00	0.00 ± 0.00	0.00 ± 0.00	2.35 ± 3.32	0.00 ± 0.00	0.00 ± 0.00	0.00 ± 0.00	14.62 ± 2.10	0.00 ± 0.00	0.00 ± 0.00	0.00 ± 0.00
C_2_	Ethanol	40.11 ± 38.52	29.14 ± 24.51	69.71 ± 31.24	6.26 ± 0.77	45.88 ± 6.69	35.43 ± 8.49	32.58 ± 7.84	15.26 ± 0.62	29.27 ± 5.10	35.32 ± 49.61	46.95 ± 7.76	22.07 ± 28.52
	Acetaldehyde	16.86 ± 0.44	16.59 ± 14.32	17.33 ± 12.53	6.14 ± 0.61	19.20 ± 3.60	9.48 ± 0.04	8.61 ± 1.16	9.21 ± 2.56	15.16 ± 6.08	8.17 ± 1.45	5.73 ± 0.76	9.10 ± 6.99
C_3_	1-Propanol	26.35 ± 0.63	0.00 ± 0.00	0.01 ± 0.02	61.62 ± 24.42	37.30 ± 8.31	0.00 ± 0.00	0.01 ± 0.01	79.88 ± 0.69	71.43 ± 4.54	0.00 ± 0.00	0.01 ± 0.01	22.07 ± 6.43
	2-Propanol	67.45 ± 4.43	0.16 ± 0.02	0.20 ± 0.03	88.43 ± 6.65	65.18 ± 2.37	0.10 ± 0.02	0.17 ± 0.01	74.84 ± 15.38	69.11 ± 4.14	0.15 ± 0.02	0.19 ± 0.04	22.86 ± 2.41
	Propanal	57.58 ± 6.97	0.54 ± 0.22	0.64 ± 0.24	15.04 ± 1.42	58.72 ± 4.20	0.49 ± 0.26	0.45 ± 0.10	15.47 ± 4.31	25.89 ± 14.86	0.26 ± 0.04	0.57 ± 0.19	7.31 ± 2.48
	2-Propanone	35.12 ± 0.63	1.09 ± 0.08	2.12 ± 0.57	9.66 ± 7.16	34.56 ± 2.70	1.12 ± 0.07	1.46 ± 0.31	6.29 ± 1.99	23.33 ± 6.13	1.00 ± 0.05	1.36 ± 0.12	21.97 ± 2.07
	Methyl acetate	23.57 ± 0.96	0.55 ± 0.08	0.58 ± 0.03	80.53 ± 21.06	35.12 ± 8.73	0.40 ± 0.13	0.63 ± 0.07	92.38 ± 2.78	69.92 ± 2.04	0.19 ± 0.22	0.28 ± 0.03	70.93 ± 6.21
	2-Chloropropane	55.06 ± 2.31	0.09 ± 0.09	0.09 ± 0.03	82.85 ± 15.26	52.09 ± 3.02	0.02 ± 0.02	0.11 ± 0.01	89.38 ± 6.54	61.32 ± 1.14	0.08 ± 0.02	0.11 ± 0.02	54.65 ± 6.57
	Trimethylsilanol	6.85 ± 1.17	0.27 ± 0.01	0.33 ± 0.04	0.21 ± 0.00	2.88 ± 0.95	0.25 ± 0.05	0.25 ± 0.01	0.36 ± 0.22	3.71 ± 0.02	0.13 ± 0.04	0.14 ± 0.00	0.29 ± 0.11
C_4_	*n*-Butanal	0.73 ± 0.40	0.03 ± 0.04	0.03 ± 0.04	0.29 ± 0.23	1.50 ± 1.02	0.03 ± 0.05	0.01 ± 0.00	0.72 ± 0.67	7.12 ± 4.48	0.02 ± 0.02	0.07 ± 0.05	5.93 ± 3.65
	2-Methylpropanal	7.81 ± 1.70	n.d.	0.00 ± 0.00	3.01 ± 2.75	18.20 ± 8.03	n.d.	0.33 ± 0.47	6.33 ± 4.37	34.94 ± 14.78	0.00 ± 0.00	0.00 ± 0.00	49.87 ± 3.36
	2-Methyl-2-propanol	12.21 ± 0.20	0.11 ± 0.01	0.11 ± 0.01	2.46 ± 2.25	6.50 ± 2.96	0.06 ± 0.01	0.11 ± 0.00	4.92 ± 4.20	11.37 ± 2.00	0.06 ± 0.02	0.09 ± 0.00	43.62 ± 16.88
	Methacroleine	4.06 ± 0.44	0.04 ± 0.02	0.12 ± 0.03	1.53 ± 0.38	8.69 ± 3.76	0.04 ± 0.05	0.08 ± 0.00	1.87 ± 0.76	23.14 ± 1.61	0.14 ± 0.02	0.19 ± 0.01	5.62 ± 7.72
	Methyl vinyl ketone	0.84 ± 0.46	0.02 ± 0.01	0.06 ± 0.06	0.07 ± 0.01	2.99 ± 1.87	0.05 ± 0.07	0.14 ± 0.09	0.07 ± 0.08	11.19 ± 2.00	0.10 ± 0.04	0.17 ± 0.00	2.58 ± 2.97
	Vinyl acetate	0.53 ± 0.42	0.12 ± 0.07	0.04 ± 0.03	1.74 ± 2.38	2.49 ± 1.39	0.01 ± 0.01	0.05 ± 0.02	0.04 ± 0.06	10.51 ± 2.28	0.03 ± 0.01	0.05 ± 0.01	0.06 ± 0.07
C_5_	*n*-Pentane	61.12 ± 2.54	0.18 ± 0.07	0.20 ± 0.01	0.07 ± 0.01	61.61 ± 4.98	0.10 ± 0.03	0.20 ± 0.00	0.12 ± 0.05	76.24 ± 7.34	0.11 ± 0.02	0.16 ± 0.02	0.56 ± 0.40
	Isoprene	44.66 ± 4.03	0.11 ± 0.06	0.08 ± 0.01	0.10 ± 0.05	49.66 ± 4.18	0.10 ± 0.01	0.13 ± 0.01	0.10 ± 0.06	70.79 ± 4.81	0.08 ± 0.01	0.13 ± 0.00	0.34 ± 0.31
C_6_	3-Methylpentane	8.46 ± 0.55	0.08 ± 0.01	0.09 ± 0.00	0.08 ± 0.01	8.44 ± 0.63	0.01 ± 0.02	0.08 ± 0.01	0.08 ± 0.01	12.59 ± 1.22	0.03 ± 0.01	0.04 ± 0.03	4.58 ± 6.38

*n.d.* not detected

A breakthrough volume (BV) of < 5% is recommended in order to ensure that no breakthrough occurred at that sample volume [44]. Even though VVOCs directly injected on Tenax TA® might be well thermally desorbed and separated on a medium polar GC column (as described in the “Gas chromatographic selectivity for target analytes” section), the adsorption performance drops significantly when passing an air flow through the sorbent bed. The obtained breakthrough volumes vary between 10 and 76%. For 13 out of 19 analytes, the breakthrough increases with increasing flow rate and sampling volume, e.g., regarding 2-methylpropanal, methacroleine, methyl vinyl ketone, vinyl acetate, and 3-methylpentane. There are just few compounds with a BV < 5% on Tenax TA®. The GCBs Carbograph 5TD and Carbopack X showed a BV < 1% for nearly all substances. Formic acid was not detected on the back-up tubes of all GCB adsorbents due to the inadequate analytical process (see “Gas chromatographic selectivity for target analytes” section). Again, limitations occurred regarding some low molecular alcohols and aldehydes such as ethanol and acetaldehyde. 2-Methylpropanal could not be detected on Carbograph 5TD at a flow rate of 50 ml min^−1^ (2 l) and 125 ml min^−1^ (2 l), but by increasing the sampling volume (125 ml min^−1^, 4 l) with no breakthrough occurring. There are just minor differences in the breakthrough data obtained for Carbograph 5TD and Carbopack X. Nevertheless, Carbograph 5TD can be assessed as superior in comparison to Carbopack X due to a better gas chromatographic separation of the VVOC substance mixture. BVs on Carbotrap are significantly worse than those on Carbopack X and Carbograph 5TD, but better than those obtained on Tenax TA®. Even though values are < 5% for some analytes, high breakthrough (20–93%) occurs for alcohols (1-/2-propanol, 2-methyl-1-propanol), 2-methylpropanal, 2-chloropropane, and methyl acetate.

In order to reduce the risk of analyte breakthrough, the save sampling volume (SSV) for a specific analyte/sorbent combination is defined as not more than 70% of the 5% BV [45]. Facing low BV, a SSV for VVOCs on Carbograph 5TD of 2–4 l is recommended. As the total sampling volume is already small, a further reduction to 1.4 and 2.8 l, respectively, is not necessarily required.

### Calibration

Calibration of the analytical method for quantitative determination of VVOCs (C_3_–C_6_) in indoor air was performed for the VVOC target analytes as liquid standards in methanol. A low concentration range from 0.0005 to 0.005 mg ml^−1^ and a high concentration range from 0.01 to 0.05 mg ml^−1^ were chosen. *n* = 10 equidistant calibration points *x*_*i*_, and *m* = 3 samples of each concentration *x*_01_ to *x*_10_ were measured. The individual working range for each target analyte was set according to the linear sector of the calibration curve. The lowest concentration could not be detected for all single VVOCs. Thus, this lowest calibration point is in the range of the limit of detection (LOD).

### Limits of detection and limits of quantitation

Limits of detection (LOD) and limits of quantitation (LOQ) of each target analyte are summarized in Table 4. Calibration ranges (μg ml^−1^) are varying in dependence of the specific substance as calculation is based on the linear area of the calibration curve. Calibration details are given in the Electronic supplementary material (ESM). With a total sampling volume of 4 l on Carbograph 5TD, the obtained LODs are equal to or lower than 3 μg m^−3^. As outlined above, ISO 16000-3 [43] is the preferred method for small aldehydes and ketones. However, the detection and quantification of these carbonyl compounds via thermal desorption with subsequent GC/MS analysis is possible with LOQs between 3 and 5 μg m^−3^. LOQs are highest for some alcohols (1-propanol, 2-methyl-2-propanol) with 7 and 8 μg m^−3^. Lower LOD and LOQ with 1 and 3 μg m^−3^ were surprisingly found for ethanol, although recovery was poor (< 1, see Fig. 3), and breakthrough volumes on Carbograph 5TD were high when sampling 2 and 4 l with a flow rate of 125 ml min^−1^ (35.43  ± 8.49% and 35.32 ± 49.61%, see Table 3). Therefore, the quantitative determination of ethanol in indoor air after sampling on Carbograph 5TD must be carefully observed in the future.

**Table 4:** Limits of detection (LOD) and limits of quantitation (LOQ) given in micrograms per cubic meter for VVOC target analytes after sampling on Carbograph 5TD with a total sampling volume of 4 l. Calibration range depends on the target analyte

C_*n*_	Compound	CAS no.	Calibration range [μg ml^−1^]	Limit of detection (LOD) [μg m^−3^]	Limit of quantitation (LOQ) [μg m^−3^]
C_2_	Ethanol	64-17-5	0.6–55.8	1	3
	Acetaldehyde	75-07-0	22.1–53.5	2	5
C_3_	1-Propanol	71-23-8	6.0–59.7	3	7
	2-Propanol	67-63-0	3.2–54.0	1	3
	Propanal	123-38-6	3.9–38.8	2	4
	2-Propanone	67-64-1	0.5–47.6	1	4
	Methyl acetate	79-20-9	2.5–62.5	1	3
	2-Chloropropane	75-29-6	2.2–37.0	1	2
	Trimethylsilanol	1066-40-6	4.7–46.6	2	4
C_4_	*n*-Butanal	123-72-8	5.9–58.5	2	5
	2-Methylpropanal	78-84-2	4.8–38.6	1	4
	2-Methyl-2-propanol	75-65-0	1.9–47.0	2	8
	Methacroleine	78-85-3	2.2–55.3	1	2
	Methyl vinyl ketone	78-94-4	3.5–34.8	1	3
	Vinyl acetate	108-05-4	2.4–60.9	1	2
C_5_	*n*-Pentane	109-66-0	2.9–47.7	1	2
	Isoprene	78-79-5	1.9–47.2	1	1
C_6_	3-Methylpentane	96-14-0	2.0–50.0	1	2

### Reaction products

The application of polymeric adsorbents and molecular sieves involves the risk of by-product formation. Hübschmann [46] identified benzene and some benzene derivatives as interfering components from Tenax, Porapak, and XAD-2/4. It is also well known that Tenax decomposes in the presence of nitrogen oxides, ozone, and other reactive compounds [47, 48]. Another known artifact in GC analysis is the formation of hemiacetals and acetals from carbonyl compounds in methanolic solution, as described for 1,1-dimethoxycyclohexane from cyclohexanone [49]. In this study, 2-butenal, which was not injected on the sorbent tubes as part of the standard solution, and methyl acetate unexpectedly appeared during some test series of different sorbents. The chemical mechanism leading to methyl acetate is unclear. It can be speculated that methyl acetate results from esterification, because acetic acid was found in trace concentrations as an impurity of carbon molecular sieves (CMS). As a potential product from the aldol condensation reaction of two acetaldehyde molecules, 2-butenal was identified (see ESM for the reaction scheme), which occurred after thermal desorption of CMS, GCBs, and Tenax TA® [49]. There is also evidence for the formation of the hemiacetal 1-methoxyethanol from acetaldehyde and methanol (see ESM for the reaction scheme), but the acetal 1,1-dimethoxyethane could not be identified. This is plausible because acidic conditions are required for the formation of acetals from hemiacetals [50]. The identification of the above mentioned reaction products was unambiguous. However, it was not possible to clearly assign if the hemiacetal reaction takes place in the methanolic standard solution or on the sorbent. In general, molecular sieves are known and applied as active materials [28]. The formation of these by-products is not considered as a severe disadvantage of the method but needs to be taken into account to avoid misinterpretation of chromatographic signals.

## Conclusions

**Fig. 4:**
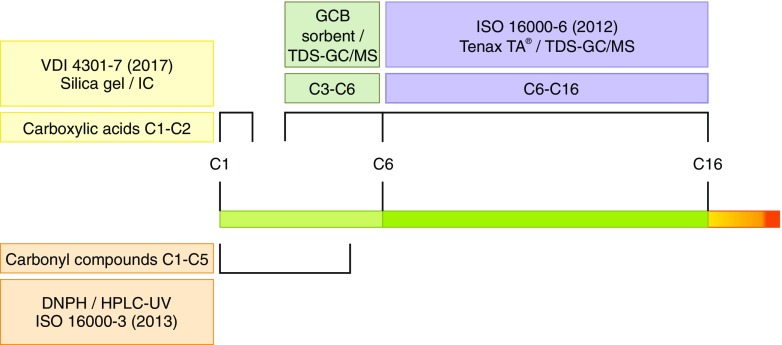
Schematic overview of available analytical methods for the quantitative determination of a large spectrum of relevant VVOCs in indoor air

By using Carbograph 5TD (20/40 mesh) as solid sorbent and a medium polar GC column, it is possible to detect VVOCs between C_3_ and C_6_ of different volatility and polarity even in trace quantities. Limitations occur for some low molecular weight compounds ≤C_3_, especially for polar substances, such as carboxylic acids (formic acid, acetic acid) and some aldehydes. At least three different analytical techniques are therefore needed to cover the large spectrum of relevant VVOCs in indoor air (see Fig. 4). This allows a significantly broadening of the analytical spectrum ≤C_6_ beyond the C_6_–C_16_ window for VOCs as defined by ISO 16000-6 [8]. Facing the definition of VVOCs in EN 16516 [11], it is important to highlight that this standard can only be applied to the specified GC column and analytical setup. As soon as the setup is changed, the definition is no longer valid. By using a medium polar GC column as in this study, substances which can fall within the class of VVOCs (regardless of the specific definition) will elute both before and after *n*-hexane (RI 534.38), e.g., isoprene (RI 516.12), 2-propanone (RI 532.02), methacroleine (RI 607.79), and methyl vinyl ketone (RI 631.49) (For calculation of retention indices and a list of retention indices of VVOC target substances, please see ESM and [51]). However, some of these substances are defined as VOCs according to EN 16516 (normative annex G), such as 2-methyl-2-propanol, *n*-butanal, and 2-methyl-1-propanol. Therefore, irrespective of any standard, a significant extension of the range of detectable and quantifiable volatile organics in indoor air was achieved in this study.

### Outlook

It is reasonable that VVOCs ≤C_3_ might need even stronger adsorbent media due to their high volatility. It is, however, still questionable if the entire range of VVOCs in indoor air C_1_–C_6_ can be determined by sampling on solid sorbents with subsequent TDS-GC/MS analysis. When handling strong sorbent media, not just water management but also the occurrence of unexpected by-products has to be considered. Furthermore, a reduction of the number of solid sorbents and analysis currently needed to cover a broad spectrum of volatile organics (VVOCs/VOCs) is desirable. The performance of multisorbent tubes will be therefore further investigated.

## Electronic supplementary material

ESM 1 (PDF 124 kb)

## References

[1] Logue JM, McKone TE, Sherman MH, Singer BC (2011). Hazard assessment of chemical air contaminants measured in residences. Indoor Air.

[2] Sahlberg B, Gunnbjörnsdottir M, Soon A, Jogi R, Gislason T, Wieslander G, Janson C, Norback D (2013). Airborne molds and bacteria, microbial volatile organic compounds (MVOC), plasticizers and formaldehyde in dwellings in three North European cities in relation to sick building syndrome (SBS). Sci Total Environ.

[3] Polizzi V, Adams A, Malysheva SV, De Saeger S, Van Peteghem C, Moretti A, Picco AM, De Kimpe N (2012). Identification of volatile markers for indoor fungal growth and chemotaxonomic classification of Aspergillus species. Fungal Biol.

[4] Tsushima S, Wargocki P, Tanabe S (2018). Sensory evaluation and chemical analysis of exhaled and dermally emitted bioeffluents. Indoor Air.

[5] Bartsch J, Uhde E, Salthammer T (2016). Analysis of odour compounds from scented consumer products using gas chromatography-mass spectrometry and gas chromatography-olfactometry. Anal Chim Acta.

[6] Morrison G, Lazaridis M, Colbeck I (2010). Chemical reactions among indoor pollutants. Human exposure to pollutants via dermal absorption and inhalation. Vol. 17. Environmental pollution.

[7] Regulation (EU) No 305/2011 of the European Parliament and of the Council of 9 March 2011 laying down harmonised conditions for the marketing of construction products and repealing Council Directive 89/106/EEC.

[8] ISO 16000-6. Indoor air—Part 6: Determination of volatile organic compounds in indoor and test chamber air by active sampling on Tenax TA® sorbent, thermal desorption and gas chromatography using MS or MS-FID. Berlin: Beuth Verlag; 2012.

[9] ISO 16000-9. Indoor air—Part 9: Determination of the emission of volatile organic compounds from building products and furnishing—emission test chamber method. Berlin: Beuth Verlag; 2008.

[10] ISO 16000-11 (2006). Indoor air—Part 11: Determination of the emission of volatile organic compounds from building products and furnishing—sampling, storage of samples and preparation of test specimens.

[11] EN 16516 (2018). Construction products: assessment of release of dangerous substances—determination of emissions into indoor air.

[12] European Collaborative Action (ECA) (2005). Harmonisation of indoor material emissions labelling systems in the EU. Inventory of existing schemes, Report No 24, Urban Air, Indoor Environment and Human Exposure.

[13] European Collaborative Action (ECA) (2012). Harmonisation framework for indoor products labelling schemes in the EU, Report No 27, Urban air, indoor environment and human exposure.

[14] European Collaborative Action (ECA) (2013). Harmonisation framework for health based evaluation of indoor emissions from construction products in the European Union using the EU-LCI concept, Report No 29, Urban air, indoor environment and human exposure.

[15] Salthammer T (2016). Very volatile organic compounds: an understudied class of indoor air pollutants. Indoor Air.

[16] Wang B, Zhao Y, Lan Z, Yao Y, Wang L, Sun H (2016). Sampling methods of emerging organic contaminants in indoor air. Trends Anal Chem.

[17] Koppmann R (2007). Volatile organic compounds in the atmosphere.

[18] Woolfenden E (2010). Sorbent-based sampling methods for volatile and semi-volatile organic compounds in air. Part 1: Sorbent-based air monitoring options. J Chromatogr A.

[19] U.S. Environmental Protection Agency (1999). Compendium Method TO-14, The determination of volatile organic compounds (VOCs) in ambient air using SUMMA(R) passivated canister sampling and gas chromatographic analysis.

[20] U.S. Environmental Protection Agency. Compendium Method TO-15, Determination of volatile organic compounds (VOCs) in air collected in specially-prepared canisters and analyzed by gas chromatography/mass spectrometry (GC/MS). In: Compendium of methods for the determination of toxic organic compounds in ambient air. 2nd ed. Cincinnati: Center for Environmental Research Information, Office of Research and Development; 1999.

[21] ASTM D5466-15 (2015). Standard test method for determination of volatile organic compounds in atmospheres (canister sampling methodology).

[22] Uhde E, Salthammer T, Uhde E (2009). Application of solid sorbents for the sampling of volatile organic compounds in indoor air. Organic indoor air pollutants. Occurrence, measurement, evaluation.

[23] ASTM D6196-15. Standard practice for choosing sorbents, sampling parameters and thermal desorption analytical conditions for monitoring volatile organic chemicals in air. West Conshohocken: ASTM International; 2015.

[24] Woolfenden E (2010). Sorbent-based sampling methods for volatile and semi-volatile organic compounds in air. Part 2: Sorbent selection and other aspects of optimizing air monitoring methods. J Chromatogr A.

[25] Dettmer K, Knobloch T, Engewald W (2000). Stability of reactive low boiling hydrocarbons on carbon based adsorbents typically used for adsorptive enchrichment and thermal desorption. Fresenius J Anal Chem.

[26] Brancaleoni E, Scovaventi M, Frattoni M, Mabilia R, Ciccioli P (1999). Novel family of multi-layer cartridges filled with a new carbon adsorbent for the quantitative determination of volatile organic compounds in the atmosphere. J Chromatogr A.

[27] Dettmer K, Bittner T, Engewald W (2001). Adsorptive enrichment and thermal desorption of low-boiling oxygenated compounds—possibilities and limitations. Chromatographia Suppl.

[28] Dettmer K, Engewald W (2002). Adsorbent materials commonly used in air analysis for adsorptive enrichment and thermal desorption of volatile organic compounds. Anal Bioanal Chem.

[29] Coeur C, Jacob V, Denis I, Foster P (1997). Decomposition of α-pinene and sabinene on solid sorbents, Tenax TA and Carboxen. J Chromatogr A.

[30] Ribes A, Carrera G, Gallego E, Roca X, Berenguer MA, Guardino X (2007). Development and validation of a method for air-quality and nuisance odors monitoring of volatile organic compounds using multi-sorbent adsorption and gas chromatography/mass spectrometry thermal desorption system. J Chromatogr A.

[31] Gallego E, Roca FJ, Perales JF, Guardino X (2010). Comparative study of the adsorption performance of a multi-sorbent bed (Carbotrap, Carbopack X, Carboxen 569) and a Tenax TA adsorbent tube for the analysis of volatile organic compounds (VOCs). Talanta.

[32] Camel V, Caude M (1995). Trace enrichment methods for the determination of organic pollutants in ambient air. J Chromatogr A.

[33] Rothweiler H, Wäger PA, Schlatter C (1991). Comparison of Tenax TA and Carbotrap for sampling and analysis of volatile organic compounds in air. Atmos Environ.

[34] Fastyn P, Kornacki W, Gierczak T, Gawłowski J, Niedzielski J (2005). Adsorption of water vapour from humid air by selected carbon adsorbents. J Chromatogr A.

[35] Fastyn P, Kornacki W, Kardas M, Gawłowski J, Niedzielski J (2003). Adsorption of water vapour from humid air in carbon molecular sieves: Carbosieve S-III and Carboxens 569, 1000 and 1001. Analyst.

[36] Brown VM, Crump DR (2013). An investigation into the performance of a multi-sorbent sampling tube for the measurement of VVOC and VOC emissions from products used indoors. Anal Methods.

[37] Patil SF, Lonkar ST (1992). Thermal desorption-gas chromatography for the determination of benzene, aniline, nitrobenzene and chlorobenzene in workplace air. J Chromatogr.

[38] Rumble J (2017). CRC handbook of chemistry and physics.

[39] McLafferty FW, Turecek F (1993). Interpretation of mass spectra.

[40] Einax JW, Zwanziger HW, Geiß S (1997). Chemometrics in environmental analysis.

[41] DIN 32645 (2008). Chemical analysis—decision limit, detection limit and determination limit under repeatability conditions—terms, methods, evaluation.

[42] VDI 4301-7 (2017). Measurement of indoor air pollution—measurement of carboxylic acids.

[43] ISO 16000-3 (2013). Indoor air—Part 3: Determination of formaldehyde and other carbonyl compounds in indoor air and test chamber air—active sampling method.

[44] U.S. Environmental Protection Agency. Compendium Method TO-17, Determination of volatile organic compounds (VOCs) in ambient air using active sampling onto sorbent tubes. In: Compendium of methods for the determination of toxic organic compounds in ambient air. 2nd ed. Cincinnati: Center for Environmental Research Information, Office of Research and Development; 1999.

[45] ISO 16017 (2001). Indoor, ambient and workplace air—sampling and analysis of volatile organic compounds by sorbent tube/thermal desorption/capillary gas chromatography—Part 1: Pumped sampling.

[46] Hübschmann H-J (2015). Handbook of GC-MS.

[47] Clausen PA, Wolkoff P (1997). Degradation products of Tenax TA formed during sampling and thermal desorption analysis: indicators of reactive species indoors. Atmos Environ.

[48] Klenø JG, Wolkoff P, Clausen PA, Wilkins CK, Pedersen T (2002). Degradation of the adsorbent Tenax TA by nitrogen oxides, ozone, hydrogen peroxide, OH radical and limonene oxidation products. Environ Sci Technol.

[49] Uhde E, Salthammer T (2007). Impact of reaction products from building materials and furnishings on indoor air quality—a review of recent advances in indoor chemistry. Atmos Environ.

[50] Sykes P (1986). A guidebook to mechanism in organic chemistry.

[51] van Den Dool H, Kratz PD (1963). A generalization of the retention index system including linear temperature programmed gas-liquid partition chromatography. J Chrom A.

