# Supplementary material for: Analytical procedure for the determination of very volatile organic compounds (C3–C6) in indoor air
Source: Anal Bioanal Chem. 2018 Mar 28;410(13):3171–83. doi: 10.1007/s00216-018-1004-z (PMC5910464; doi:10.1007/s00216-018-1004-z)
Supplement: Supplementary file 1 — (PDF 124 kb) [file 216_2018_1004_MOESM1_ESM.pdf]

Analytical and Bioanalytical Chemistry

Electronic Supplementary Material

Analytical procedure for the determination of very volatile organic compounds (C_3-C_6) in indoor air

Alexandra Schieweck, Jan Gunschera, Deniz Varol, Tunga Salthammer

Retention indices (RI)

Non-isothermal Kovats retention indices (RI) were calculated according to the definition by Van den Dool and Kratz [51]

$$RI = 100 \cdot c + 100 \frac{(t'_R)_x - (t'_R)_c}{(t'_R)_{c+1} - (t'_R)_c}$$

With c, the number of carbon atoms in the smaller n-alkane, c+1, the number of carbon atoms in the larger alkane, x, unknown RI of substance x, t'_R, retention time adjusted against dead time (t_0).

The dead time (t_0) is the retention time which the carrier gas (mobile phase) needs to pass through the GC column from injection to detection. For calculation, dead time of methanol (t_0: 4.7 min) was chosen as it was used as solvent for the VVOC standard mixture.

Adjusted retention times of n-alkanes were obtained by measuring an n-alkane series (C5-C20) with the new analytical method described. In order to include C1-C4-alkanes into the series, the injection of gaseous standards on a solid sorbent is necessary. As this technology currently lacks of reproducibility and robustness, C1-C4-alkanes could not be included. Thus, calculation of RI for the substances acetaldehyde (c: 4, c+1: 5) and n-pentane (c: 4, c+1: 6) was not possible yet. Moreover, (t_0) of methanol could not be chosen as it elutes after acetaldehyde on a medium-polar GC column which results in a negative value for $(t'_R)_x$.

Table S1 summarizes the retention times (rt) and retention indices (RI) for each target analyte. Analytes are sorted by retention time.

Table S1 Retention times (rt) and retention indices (RI) for each target analyte

Compound	CAS-no.	Formula	Rt [min]	RI
Acetaldehyde	75-07-0	CH_3CHO	3.73	n.c.
n-Pentane	109-66-0	C_5H_{12}	5.47	n.c.
Ethanol	64-17-5	C_2H_5OH	5.83	510.12
Isoprene	78-79-5	$CH_2C(CH_3)CHCH_2$	6.12	516.12
2-Chloropropane	75-29-6	$CH_3CHClCH_3$	6.28	519.42
Propanal	123-38-6	C_3H_6O	6.6	526.03
2-Propanone	67-64-1	$CO(CH_3)_2$	6.89	532.02
2-Propanol	67-63-0	$CH_3CH(OH)CH_3$	7.53	545.25
Methyl acetate	79-20-9	CH_3COOCH_3	8.06	556.20
2-Methyl-2-propanol	75-65-0	$CH_3C(CH_3)(OH)CH_3$	8.99	575.41
3-Methylpentane	96-14-0	$C_2H_5CH(CH_3)C_2H_5$	9.33	582.44
2-Methylpropanal	78-84-2	$CH_3CH(CH_3)CHO$	10.11	598.55
Methacroleine	78-85-3	$CH_2C(CH_3)CHO$	10.9	607.79
Vinyl acetate	108-05-4	$CH_3C(O)OCHCH_2$	11.48	614.07
1-Propanol	71-23-8	$CH_3(CH_2)_2OH$	11.9	618.61
n-Butanal	123-72-8	C_4H_8O	12.95	629.98
Methyl vinyl ketone	78-94-4	$CH_3C(O)CHCH_2$	13.09	631.49
Trimethylsilanol	1066-40-6	$C_3H_{10}OSi$	14.33	644.91

n.c. not calculated

Reaction schemes

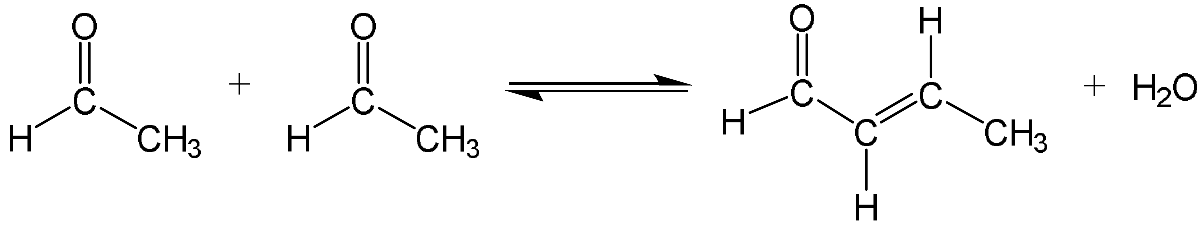

Scheme S1 Formation of 2-butenal from aldol condensation of acetaldehyde

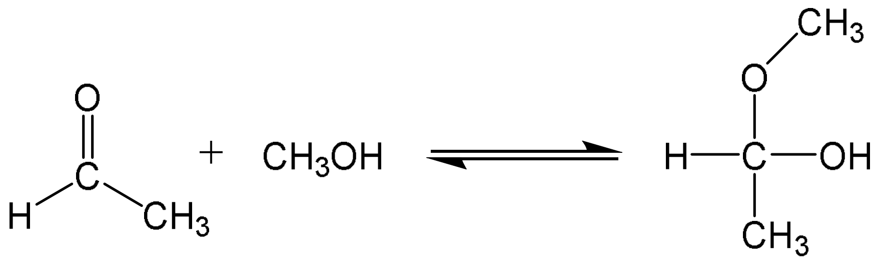

Scheme S2 Formation of the hemiacetal 1-methoxy ethanol from the reaction of acetaldehyde and methanol
